# Cardiorespiratory coupling during high-frequency oscillatory ventilation with volume guarantee in extremely preterm infants

**DOI:** 10.1007/s00431-026-07429-w

**Published:** 2026-09-23

**Authors:** Kamal Ali, Abdulaziz Homedi, Musab Alshareef, Mohanned Alrahili, Ibrahim Ali, Saif Alsaif, Mahesh Nanjundappa, Theodore Dassios, Anne Greenough

**Affiliations:** 1 Neonatal Intensive Care Department, King Abdulaziz Medical City, Riyadh, Saudi Arabia; 2 Neonatal Intensive Care Centre, King’s College Hospital, 4 Floor, Golden Jubilee Wing, Denmark Hill, London, SE5 9RS UK; 3 King Abdullah International Medical Research Centre, Riyadh, Saudi Arabia; 4 Department of Women and Children’s Health, School of Life Course Sciences, Faculty of Life Sciences and Medicine, King’s College London, London, UK; 5 College of Medicine, King Saud Bin Abdulaziz University for Health Sciences, Riyadh, Saudi Arabia

**Keywords:** High-frequency oscillatory ventilation, Volume guarantee, Targeted neonatal echocardiography, Cardiorespiratory interaction, Preterm infants

## Abstract

This study aims to evaluate associations between PCA-derived cardiovascular components and respiratory burden during high-frequency oscillatory ventilation with volume guarantee (HFOV-VG). This retrospective cohort study included infants born before 30 weeks of gestation who received HFOV-VG and underwent targeted neonatal echocardiography during respiratory support. Echocardiography assessed ventricular systolic function, cardiac output, myocardial deformation, and pulmonary vascular loading. Principal component analysis (PCA) derived integrated cardiovascular components. Multivariate regression models evaluated associations with respiratory support, haemodynamic support, and clinical outcomes, including mortality, moderate-to-severe bronchopulmonary dysplasia (BPD), and the composite outcome of death or moderate-to-severe BPD. Sixty-two infants were included (gestational age 26.0 [25.0–27.0] weeks; birth weight 860 [700–1000] g). Median FiO₂ during HFOV-VG was 0.35 (0.31–0.52). Right ventricular strain demonstrated the strongest association with FiO₂ (rho = 0.53, *q* < 0.001). The first principal component (PC1), representing a cardiopulmonary loading component characterised by less favourable ventricular performance, reduced cardiac output, altered myocardial deformation, and increased septal loading, explained 65.9% of echocardiographic variance. Each one standard deviation increase in PC1 was associated with a 0.60 standard deviation increase in FiO₂ (95% CI 0.38–0.82; *p* < 0.001). Higher PC1 was independently associated with mortality (OR 3.01, 95% CI 1.44–6.29; *p* = 0.004), higher peak vasoactive–inotropic score, longer inotropic support, increased odds of inotrope use, and lower odds of haemodynamically significant patent ductus arteriosus (all *p* < 0.05).

*Conclusion*: In preterm infants receiving HFOV-VG, the first cardiovascular principal component was independently associated with oxygenation burden, haemodynamic support requirements, and mortality. PCA-derived cardiovascular assessment may complement conventional respiratory assessment during HFOV-VG.

**Table:** 

**What is Known:** • *Oxygenation during high-frequency oscillatory ventilation is influenced by both respiratory and cardiovascular factors*. • *Targeted neonatal echocardioghraphy can assess multiple aspects of cardiovascular function, but most studies have evaluated individual haemodynamic variables rather than integrated cardiovascular profiles*.
**What is New:** • *Exploratory principal component analysis identified a cardiovascular component independently associated with oxygen requirements during HFOV-VG*. • *The first principal component was independently associated with oxygenation burden, haemodynamic support requirements, and mortality, supporting an integrated cardiorespiratory approach to physiological assessment during HFOV-VG*.

**Supplementary Information:**

The online version contains supplementary material available at https://doi.org/10.1007/s00431-026-07429-w.

## Introduction

Preterm infants frequently require invasive respiratory support because of surfactant deficiency, lung immaturity, impaired gas exchange, and evolving pulmonary vascular disease. Cardiovascular adaptation after birth also influences oxygenation, systemic perfusion, and clinical stability. This transition involves major changes in pulmonary blood flow, ventricular loading, and myocardial performance, which may be particularly pronounced in preterm infants [1, 2].

Cardiorespiratory interaction is especially relevant during invasive ventilation. Increased pulmonary vascular resistance, right ventricular pressure loading, impaired ventricular performance, and reduced cardiac output may compromise pulmonary perfusion and oxygenation. Conversely, respiratory support can alter preload, afterload, and ventricular function through changes in intrathoracic pressure and pulmonary haemodynamics. These bidirectional interactions suggest that oxygen requirements may reflect cardiovascular status and pulmonary disease [2, 3].

High-frequency oscillatory ventilation with volume guarantee (HFOV-VG) is increasingly used in preterm infants with severe respiratory failure [4, 5]. By targeting a predefined oscillatory tidal volume, HFOV-VG aims to optimise carbon dioxide clearance while limiting lung-volume instability and ventilator-induced injury. It can provide effective gas exchange and ventilatory efficiency at relatively low tidal volumes [6–8]. Nevertheless, variability in oxygen requirements persists, suggesting that factors beyond respiratory mechanics may contribute to oxygenation burden.

Targeted neonatal echocardiography provides bedside assessment of ventricular performance, cardiac output, pulmonary vascular loading, and ventricular interaction [9]. Two-dimensional speckle-tracking echocardiography further evaluates myocardial deformation and may identify functional changes not detected by conventional indices [10, 11].

Previous studies have generally examined individual echocardiographic markers, although neonatal cardiovascular status reflects interactions among multiple haemodynamic domains. Individual measurements may therefore provide an incomplete representation of the cardiovascular contribution to respiratory failure. We aimed to determine whether a PCA-derived cardiovascular component was associated with oxygenation burden and adverse clinical outcomes in infants born before 30 weeks of gestation receiving HFOV-VG.

## Methods

### Study design, setting, and participants

This retrospective observational study was conducted between November 2024 and February 2026 in the neonatal intensive care unit (NICU) of King Abdulaziz Medical City, Riyadh, Saudi Arabia. The study included preterm infants born before 30 weeks of gestation who received high-frequency oscillatory ventilation with volume guarantee (HFOV-VG) during the early neonatal period.

Eligible infants were required to have minute-level ventilator recordings available during HFOV-VG and to have undergone a comprehensive targeted neonatal echocardiographic assessment during the corresponding period of respiratory support. Infants with major congenital anomalies, chromosomal abnormalities, or congenital heart disease other than a patent ductus arteriosus or a small atrial-level shunt were excluded.

Related physiological analyses from an overlapping cohort of preterm infants receiving HFOV-VG have been published previously [12]. The present study addressed a distinct research question by evaluating contemporaneous targeted neonatal echocardiographic and ventilator findings and examining the associations of individual echocardiographic measures and PCA-derived cardiovascular components with oxygenation burden, haemodynamic support requirements and clinical outcomes.

The study was approved by the Institutional Review Board of King Abdullah International Medical Research Center (KAIMRC). Owing to its retrospective design and the use of anonymised routinely collected clinical data, the requirement for informed parental consent was waived.

### Respiratory and cardiovascular assessment

All infants were ventilated using the Dräger Babylog VN600 (Dräger Medical, Lübeck, Germany). HFOV-VG was the unit’s standard first-line invasive ventilation mode for preterm infants with respiratory distress syndrome, generally initiated within 6 h after birth rather than as rescue therapy following conventional ventilation failure. All infants received surfactant before lung recruitment. An open-lung strategy was then used, with mean airway pressure adjusted stepwise according to oxygenation response and clinical and radiographic lung inflation, and subsequently individualised to maintain recruitment while avoiding overdistension. Volume guarantee targeted a high-frequency tidal volume of approximately 1.5–2.0 mL/kg, while frequency was generally maintained at 15–17 Hz and adjusted according to blood gases and clinical response. FiO₂, amplitude, mean airway pressure, frequency, measured and set tidal volumes, and DCO₂ were recorded electronically at minute-level resolution and summarised for each infant.

Targeted neonatal echocardiography was performed during HFOV-VG within the first 48 h after birth by neonatologists certified in neonatal haemodynamic assessment, using a Vivid ultrasound platform with a neonatal multifrequency transducer (GE HealthCare, Milwaukee, WI, USA). Examinations followed established recommendations and were contemporaneous with the analysed ventilator data [9].

LVEF and LVFS were derived from parasternal long-axis M-mode measurements obtained perpendicular to the interventricular septum and posterior wall at the mitral leaflet tips; LVEF was calculated using the Teichholz formula. LVO and RVO were calculated from the respective outflow-tract diameter, Doppler velocity–time integral and heart rate and indexed to body weight. TAPSE was measured using M-mode, and RV FAC from end-diastolic and end-systolic RV areas, in the apical four-chamber view.

PAAT and RVET were measured from pulsed-wave Doppler recordings in the proximal pulmonary artery. PAAT/RVET was used as a surrogate of pulmonary vascular resistance. End-systolic eccentricity index was measured in the parasternal short-axis view at the papillary-muscle level to assess septal configuration and right ventricular pressure loading.

Two-dimensional speckle-tracking was performed using optimal-quality apical four-chamber images analysed offline with the commercially available LV and RV Auto Strain packages (GE HealthCare, Milwaukee, WI, USA). Automated endocardial borders were manually adjusted when required, with reference points placed at the basal septal, basal lateral or free-wall annular, and apical regions. LV strain represented four-chamber longitudinal strain and RV strain represented RV free-wall longitudinal strain, measured according to established neonatal methodologies [10, 11, 13].

### Study variables and outcomes

Mean FiO₂ during HFOV-VG was the primary physiological outcome and measure of oxygenation burden. Because all infants were managed using a standardised oxygen saturation target of 90–95%, differences in mean FiO₂ reflected differences in oxygen requirements. Cardiorespiratory coupling was evaluated by examining associations between mean FiO₂ and contemporaneous individual echocardiographic measures and PCA-derived cardiovascular components.

Data on vasoactive and inotropic treatment included medication exposure, maximum doses recorded during the first 48 h after birth, and total treatment duration. Dopamine, dobutamine, epinephrine, norepinephrine, milrinone and vasopressin were included. Haemodynamic support intensity was quantified using a modified vasoactive–inotropic score (VIS) based on Gaies et al. [14]:

$$VIS=dopamine+dobutamine+100\times epinephrine+100\times norepinephrine+10\times milrinone+10\times vasopres\sin$$

Vasopressin doses were recorded in mIU/kg/min. Peak VIS corresponded to the maximum vasoactive support during the first 48 h but was not time-locked to the exact echocardiogram. hsPDA status was determined from the same echocardiographic examination and defined as an Iowa PDA Severity Score ≥ 6 [15].

Mortality before hospital discharge was the primary clinical outcome. Secondary outcomes included moderate-to-severe BPD, defined as Jensen grades 2–3 [16]; death or moderate-to-severe BPD; pneumothorax; postnatal corticosteroid exposure; major intraventricular haemorrhage, defined as grade III–IV [17]; and duration of invasive ventilation.

### Statistical analysis

Statistical analyses were performed using SPSS version 31 (IBM Corp., Armonk, NY, USA).

Continuous variables were assessed for normality using the Shapiro–Wilk test. As most were non-normally distributed, they are presented as median (interquartile range [IQR]); categorical variables are presented as number and percentage.

Associations between individual echocardiographic variables and oxygenation/exposure burden during HFOV-VG were assessed using Spearman rank correlation coefficients with 95% confidence intervals. P values were adjusted for multiple testing using the Benjamini–Hochberg false-discovery-rate method, and adjusted q values are reported [18]. Correlations were classified by absolute magnitude as negligible (0.00–0.09), weak (0.10–0.39), moderate (0.40–0.69), strong (0.70–0.89), or very strong (0.90–1.00) [19].

Principal component analysis (PCA) was used as an unsupervised data-reduction method to summarise 10 correlated echocardiographic variables representing ventricular performance, myocardial deformation, cardiac output, and pulmonary vascular loading [20]. Variables were standardised before analysis, and PCA was conducted as a complete-case analysis among 58 infants with data available for all variables. Sampling adequacy and suitability of the correlation matrix were assessed using the Kaiser–Meyer–Olkin statistic and Bartlett’s test of sphericity, respectively [21, 22]. Components were interpreted according to their dominant loadings and physiological characteristics. PC1 was selected for subsequent analyses because it explained the greatest proportion of total variance. Higher PC1 scores represented a cardiovascular pattern characterised by less favourable ventricular performance, lower cardiac output, altered myocardial deformation, and greater cardiopulmonary loading.

Multivariate linear regression evaluated associations between PC1 and continuous respiratory and haemodynamic variables, including mean FiO₂, mean airway pressure, oscillatory amplitude, oscillatory frequency, DCO₂, peak vasoactive–inotropic score, and duration of inotropic support. Multivariate logistic regression evaluated associations with mortality, moderate-to-severe bronchopulmonary dysplasia, pneumothorax, postnatal corticosteroid exposure, major intraventricular haemorrhage, haemodynamically significant patent ductus arteriosus, and inotrope exposure. All models were adjusted for gestational age and birth weight. Results are presented as standardised beta coefficients or odds ratios (ORs) with 95% confidence intervals, as appropriate. The incremental contribution of PC1 was assessed using adjusted *R*^2^ for linear models and Nagelkerke pseudo-R^2^ for logistic models [23].

For tertile analyses, the 58 infants included in the PCA were categorised by PC1 score into low, intermediate, and high-burden groups. Trends across tertiles were assessed using Spearman rank correlation for continuous variables and logistic regression for binary outcomes.

Receiver operating characteristic analysis evaluated discrimination of mortality by PC1 alone and by models incorporating gestational age, birth weight, and FiO₂. Performance was quantified using the area under the curve (AUC) with 95% confidence intervals.

All tests were two-sided, and *p* < 0.05 was considered statistically significant.

## Results

### Cohort characteristics and cardiorespiratory profile during HFOV-VG

The study included 62 preterm infants with a median gestational age of 26.0 (25.0–27.0) weeks and birth weight of 860 (700–1000) g. All infants had respiratory distress syndrome and received surfactant. Mortality occurred in 10 (16.1%), moderate-to-severe bronchopulmonary dysplasia (BPD) in 34 (54.8%), and death or moderate-to-severe BPD in 42 (67.7%) infants (Table 1).

**Table 1:** Baseline demographic, perinatal and clinical outcome characteristics of the cohort

Variable	Value
Gestational age, weeks	26.0 (25.0–27.0)
Birth weight, g	860 (700–1000)
Male sex	27/62 (43.5%)
Antenatal steroid exposure	47/62 (75.8%)
Caesarean delivery	37/62 (59.7%)
Respiratory distress syndrome	62/62 (100%)
Surfactant administration	62/62 (100%)
Mortality	10/62 (16.1%)
Moderate-to-severe BPD	34/62 (54.8%)
Death or moderate-to-severe BPD	42/62 (67.7%)
Pneumothorax	4/62 (6.5%)

Values are presented as median (interquartile range) or *n*/*N* (%), as appropriate. *BPD*, bronchopulmonary dysplasia

During HFOV-VG, median FiO₂ was 0.35 (0.31–0.52), mean airway pressure was 10.6 (9.8–11.8) mbar, and invasive ventilation lasted 9.5 (6.0–19.2) days. Inotropic support was administered to 32 infants (51.6%), and 27 (43.5%) had a haemodynamically significant patent ductus arteriosus. Echocardiography was performed during HFOV-VG within the first 48 h after birth (Supplementary Table S1).

### Associations between echocardiographic markers and oxygenation burden during HFOV-VG

**Fig. 1:**
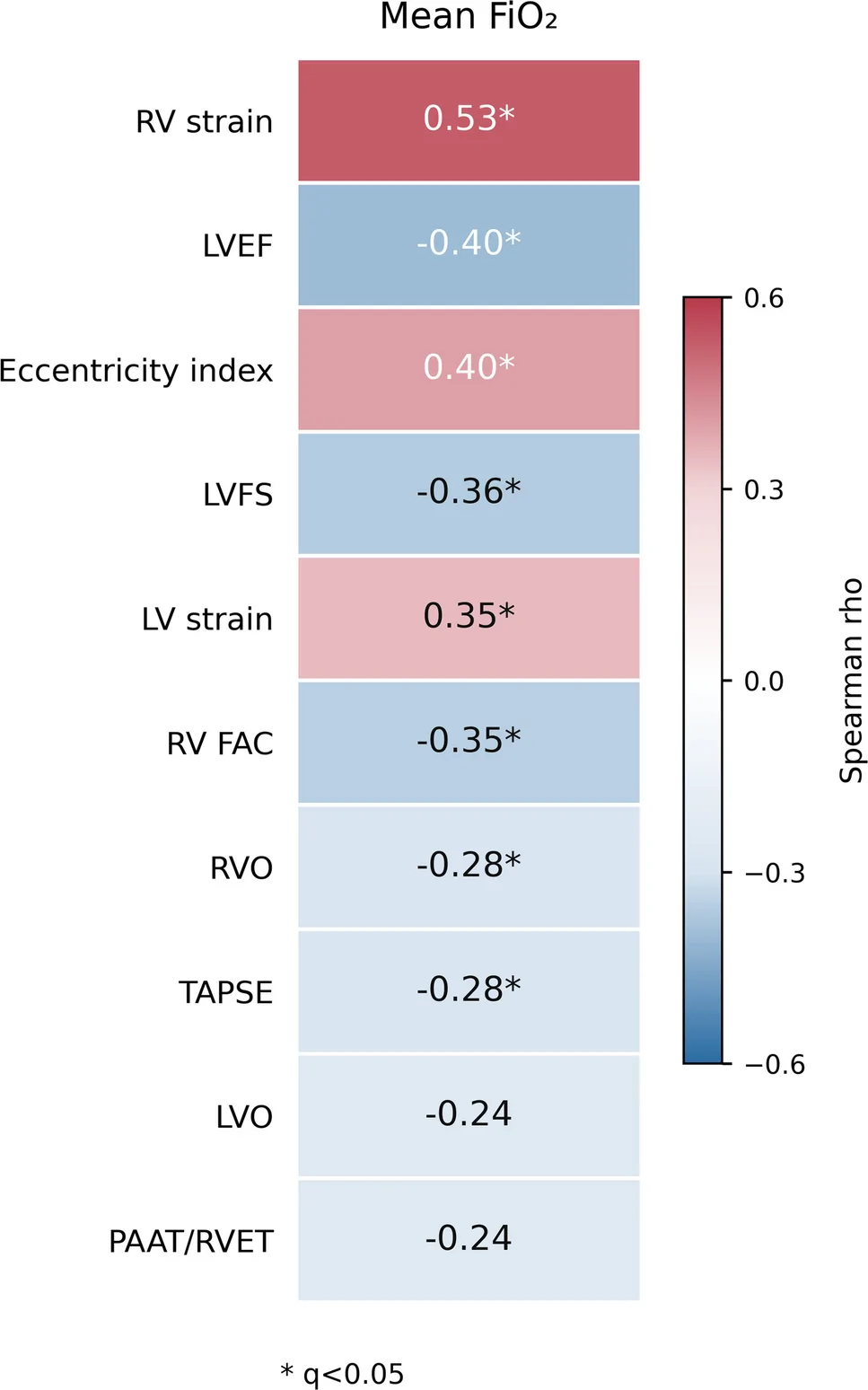
Heat map of correlations between echocardiographic measures and oxygenation burden during HFOV-VG. Cell values are Spearman rho coefficients. Red and blue indicate positive and negative correlations, respectively, with colour intensity reflecting correlation magnitude. *q < 0.05. FAC, fractional area change; LV, left ventricular; LVEF, left ventricular ejection fraction; LVFS, left ventricular fractional shortening; LVO, left ventricular output; PAAT/RVET, pulmonary artery acceleration time-to-right ventricular ejection time ratio; RV, right ventricular; RVO, right ventricular output; TAPSE, tricuspid annular plane systolic excursion

RV strain demonstrated the largest association with FiO₂, with a moderate positive correlation (rho = 0.53, *q* < 0.001). LVEF and eccentricity index also demonstrated moderate correlations, whereas the significant associations involving LVFS, LV strain, RV FAC, RVO and TAPSE were weak. Higher FiO₂ was associated with less negative ventricular strain, lower measures of ventricular performance and output, and a higher eccentricity index. LVO and PAAT/RVET were not significantly associated with FiO₂ after false-discovery-rate correction (Fig. 1 and Supplementary Table S2).

### PCA-derived cardiovascular components

Principal component analysis identified three cardiovascular components representing exploratory physiological patterns (Table 2). PCA suitability was confirmed by a Kaiser–Meyer–Olkin statistic of 0.883 and a significant Bartlett’s test of sphericity (*χ*^2^ = 522.62, df = 45, *p* < 0.001). The first principal component (PC1) was dominant, accounting for 65.9% of total echocardiographic variance. PC1 was characterised by lower ventricular systolic performance and cardiac output indices (LVO, LVEF, LVFS, RVO, TAPSE and RV FAC), together with numerically higher (less negative) myocardial strain values and a higher eccentricity index, representing an integrated cardiopulmonary functional and loading pattern.

**Table 2:** Principal component analysis of cardiovascular patterns during HFOV-VG

Component	Dominant contributing variables	Variance explained (%)	Physiological interpretation
PC1: Integrated cardiopulmonary loading component	Lower LVO, LVEF, LVFS, RVO, TAPSE and RV FAC; higher LV strain, RV strain and eccentricity index	65.9	Less favourable ventricular performance, lower cardiac output, altered myocardial deformation0 and greater cardiopulmonary loading
PC2: Pulmonary vascular loading component	Lower PAAT/RVET and higher eccentricity index	11.4	Pattern dominated by pulmonary vascular loading and interventricular septal configuration
PC3: Myocardial deformation component	Higher LV strain and RV strain	5.7	Pattern dominated by altered longitudinal myocardial deformation

PCA was conducted in 58 infants with complete data for all 10 echocardiographic variables. The Kaiser–Meyer–Olkin statistic was 0.883, and Bartlett’s test of sphericity was significant (*χ*^2^ = 522.62, df = 45, *p* < 0.001). PC1–PC3 collectively explained 83.0% of the total variance. *FAC* fractional area change, *LV* left ventricular, *LVEF* left ventricular ejection fraction, *LVFS* left ventricular fractional shortening, *LVO* left ventricular output, *PAAT/RVET* pulmonary artery acceleration time-to-right ventricular ejection time ratio, *PC* principal component, *RV* right ventricular, *RVO* right ventricular output, *TAPSE* tricuspid annular plane systolic excursion

The second principal component (PC2) accounted for 11.4% of the variance and predominantly reflected pulmonary vascular loading and interventricular septal configuration. The third principal component (PC3) accounted for 5.7% of the variance and predominantly reflected myocardial deformation. Together, the first three components explained 83.0% of the variance within the echocardiographic dataset.

### Associations between the integrated cardiovascular component and respiratory and haemodynamic support requirements

After adjustment for gestational age and birth weight, each one-standard-deviation increase in PC1 was associated with a 0.60-standard-deviation increase in mean FiO₂ (95% CI 0.38–0.81; *p* < 0.001), increasing the adjusted *R*^2^ from 0.013 to 0.353 (Table 3). PC1 was not significantly associated with mean airway pressure, oscillatory amplitude, oscillatory frequency, or DCO₂.

**Table 3:** Incremental contribution of the integrated cardiovascular phenotype (PC1) to respiratory and haemodynamic support requirements during HFOV-VG

Endpoint	Adjusted effect per 1 SD higher PC1 (95% CI)	*p* value	Incremental R^2^ from PC1
Respiratory requirements			
Mean FiO₂ (%)	Standardized *β* 0.60 (0.38 to 0.81)	< 0.001	+ 0.340
Mean MAP (mbar)	Standardized *β* 0.21 (− 0.05 to 0.48)	0.110	+ 0.045
Oscillatory amplitude (mbar)	Standardized *β* 0.00 (− 0.26 to 0.26)	0.984	+ 0.000
Oscillatory frequency (Hz)	Standardized *β* 0.16 (− 0.10 to 0.43)	0.221	+ 0.026
DCO₂	Standardized *β* 0.11 (− 0.07 to 0.29)	0.230	+ 0.011
Haemodynamic support requirements			
Peak VIS	Standardized *β* 0.40 (0.16 to 0.65)	0.001	+ 0.156
Duration of inotropes (days)	Standardized *β* 0.31 (0.05 to 0.57)	0.020	+ 0.092
Any inotrope exposure	Adjusted OR 3.55 (1.17 to 10.77)	0.025	+ 0.205*
hsPDA	Adjusted OR 0.32 (0.12 to 0.82)	0.018	+ 0.176*

PC1 denotes the integrated adverse cardiopulmonary loading phenotype. *VIS* vasoactive-inotropic score, *hsPDA* haemodynamically significant patent ductus arteriosus. *Pseudo-*R*^2^ values are reported for binary outcomes

Higher PC1 scores were independently associated with greater haemodynamic support requirements, including higher peak VIS, longer inotropic support, and increased odds of inotrope use (adjusted OR 3.55, 95% CI 1.17–10.77; *p* = 0.025). Conversely, higher PC1 scores were associated with lower odds of hsPDA (adjusted OR 0.32, 95% CI 0.12–0.82; *p* = 0.018).

### Clinical outcomes according to cardiovascular component burden

Increasing PC1 burden was associated with higher oxygen requirements, peak VIS, duration of inotropic support, and frequency of inotrope use (Table 4).

**Table 4:** Clinical implications of increasing cardiovascular phenotype burden during HFOV-VG

Variable or model	Low burden (*n* = 19)	Intermediate burden (*n* = 19)	High burden (*n* = 20)	*p* trend	Adjusted estimate per 1 SD higher PC1 or AUC (95% CI)
Respiratory and haemodynamic support burden					
Mean FiO₂, %	31.1 (26.9–36.7)	34.9 (30.5–39.3)	40.3 (34.5–50.6)	< 0.001	*β* 0.60 (0.38 to 0.82); *p* < 0.001
Mean MAP, mbar	10.6 (9.8–11.0)	10.1 (9.6–10.9)	11.0 (10.3–12.9)	0.163	*β* 0.21 (− 0.06 to 0.48); *p* = 0.123
DCO₂	38.5 (29.4–67.3)	34.0 (31.5–41.7)	36.2 (29.8–52.8)	0.736	*β* 0.11 (− 0.08 to 0.29); *p* = 0.251
Invasive ventilation, days	9.0 (4.5–16.0)	13.0 (6.0–21.0)	7.5 (6.8–13.2)	0.919	*β* − 0.20 (− 0.46 to 0.06); *p* = 0.134
Peak VIS	0.0 (0.0–9.0)	0.0 (0.0–16.0)	40.0 (10.0–127.5)	< 0.001	*β* 0.52 (0.28 to 0.75); *p* < 0.001
Inotrope duration, days	0.0 (0.0–1.5)	0.0 (0.0–2.0)	3.0 (1.8–5.2)	< 0.001	*β* 0.40 (0.15 to 0.64); *p* = 0.002
Clinical outcomes					
Mortality	1/19 (5.3)	0/19 (0.0)	9/20 (45.0)	0.007	OR 3.01 (1.44–6.29); *p* = 0.004
Moderate-to-severe BPD	10/19 (52.6)	12/19 (63.2)	10/20 (50.0)	0.859	OR 0.63 (0.34–1.14); *p* = 0.126
Death/moderate-to-severe BPD	10/19 (52.6)	12/19 (63.2)	18/20 (90.0)	0.015	OR 2.12 (0.69–6.51); *p* = 0.190
Pneumothorax	1/19 (5.3)	0/19 (0.0)	3/20 (15.0)	0.247	OR 6.61 (1.41–30.89); *p* = 0.016
Postnatal steroids	8/19 (42.1)	11/19 (57.9)	9/20 (45.0)	0.868	OR 0.62 (0.33–1.15); *p* = 0.128
Major IVH	3/19 (15.8)	6/19 (31.6)	5/20 (25.0)	0.512	OR 1.02 (0.54–1.93); *p* = 0.958
Inotrope use	6/19 (31.6)	6/19 (31.6)	16/20 (80.0)	0.004	OR 3.65 (1.20–11.10); *p* = 0.022
hsPDA	12/19 (63.2)	7/19 (36.8)	8/20 (40.0)	0.155	OR 0.31 (0.12–0.82); *p* = 0.018
Mortality prediction models					
Model					AUC (95% CI)
FiO₂ alone					0.75 (0.57–0.90)
PC1 alone					0.86 (0.69–0.97)
PC1 + FiO₂					0.83 (0.64–0.96)
GA + birth weight					0.78 (0.59–0.92)
GA + birth weight + PC1					0.85 (0.69–0.97)
GA + birth weight + PC1 + FiO₂					0.84 (0.68–0.97)

Values are median (IQR) or *n*/*N* (%). Adjusted estimates are standardised *β* coefficients for continuous outcomes and odds ratios for binary outcomes. *PC1* principal component 1, *AUC* area under the receiver operating characteristic curve, *BPD* bronchopulmonary dysplasia, *DCO₂* carbon dioxide diffusion coefficient, *FiO₂* fraction of inspired oxygen, *GA* gestational age, *hsPDA* haemodynamically significant patent ductus arteriosus, *IVH* intraventricular haemorrhage, *MAP* mean airway pressure, *VIS* vasoactive–inotropic score

Mortality was concentrated in the high-burden group, occurring in 1/19 (5.3%), 0/19 (0%), and 9/20 (45.0%) infants in the low-, intermediate-, and high-burden groups, respectively (p for trend = 0.007). Each one-standard-deviation increase in PC1 was associated with higher adjusted odds of mortality (OR 3.01, 95% CI 1.44–6.29; *p* = 0.004). PC1 demonstrated good discrimination for mortality (AUC 0.86, 95% CI 0.69–0.97). The AUC was 0.78 for gestational age and birth weight and 0.85 after inclusion of PC1.

Higher PC1 scores were also independently associated with pneumothorax and lower odds of hsPDA. Although the composite outcome of death or moderate-to-severe BPD was more frequent in the high-burden group (52.6%, 63.2%, and 90.0% across tertiles; p for trend = 0.015), its adjusted association with PC1 was not statistically significant. PC1 was not independently associated with moderate-to-severe BPD, postnatal corticosteroid exposure, or major IVH.

## Discussion

In this study of preterm infants born before 30 weeks of gestation receiving HFOV-VG, a PCA-derived cardiovascular component was independently associated with oxygenation burden. Several echocardiographic measures reflecting ventricular performance, longitudinal myocardial deformation, cardiac output, and ventricular loading were associated with FiO₂ requirements, with RV free-wall longitudinal strain demonstrating the strongest correlation. PCA identified a dominant cardiopulmonary loading component that explained 65.9% of the echocardiographic variance and remained independently associated with oxygen requirements after adjustment for gestational age and birth weight. Higher PC1 scores were also associated with greater haemodynamic support requirements and mortality.

We selected FiO₂ as the primary respiratory endpoint because the objective was to evaluate oxygenation burden and cardiorespiratory coupling rather than overall respiratory failure severity. Unlike the oxygenation index, FiO₂ does not incorporate clinician-selected mean airway pressure and, under the standardised oxygen-saturation target used in this cohort, provided a pragmatic measure of oxygen requirement. Nevertheless, FiO₂ remains influenced by pulmonary disease, cardiovascular physiology, and clinical management and should not be regarded as a cardiovascular-specific measure. Consistent with a contribution from cardiorespiratory interaction, the PCA-derived cardiovascular component was independently associated with FiO₂ but not with the measured ventilator variables. These findings suggest that oxygen requirements during HFOV-VG may reflect both pulmonary and cardiovascular factors and that integrated haemodynamic assessment could provide complementary information beyond individual echocardiographic measurements.

The association between PC1 and oxygen requirements is physiologically plausible. Among individual echocardiographic measures, less-negative RV free-wall strain showed the strongest correlation with FiO₂, while higher eccentricity index was also associated with greater oxygen requirements. These findings suggest contributions from pulmonary vascular loading, altered RV mechanics, and ventricular interaction. Increased pulmonary vascular resistance may cause RV pressure loading, septal flattening, altered ventricular interdependence, and impaired pulmonary perfusion [24–26]. Through ventricular interaction, RV pressure loading may also affect LV performance and overall cardiorespiratory status [27]. M-mode-derived LVEF and LVFS predominantly reflect radial cavity dimensions, eccentricity index reflects septal geometry, and strain assesses longitudinal myocardial deformation. These measures therefore capture distinct but complementary aspects of ventricular mechanics. Less-negative strain should not necessarily be interpreted as intrinsic myocardial dysfunction and may instead represent load-dependent adaptation or compensation for altered septal and radial mechanics [10, 11]. Although PAAT/RVET was not significantly correlated with FiO₂ after false-discovery-rate correction, it remains a recognised surrogate of pulmonary vascular loading in neonatal and paediatric populations [28].

A novel aspect of this study was the identification of a composite cardiovascular component incorporating ventricular systolic performance, myocardial deformation, cardiac output, and pulmonary vascular loading. Neonatal haemodynamics are inherently multidimensional, and many echocardiographic variables reflect overlapping aspects of cardiovascular physiology rather than discrete physiological processes [1–3, 29]. Principal component analysis demonstrated that these measurements clustered into a dominant component accounting for approximately two-thirds of the variability within the echocardiographic dataset. This component was characterised by less favourable conventional measures of ventricular performance, reduced cardiac output, altered longitudinal myocardial deformation, and increased septal loading, representing an integrated cardiopulmonary loading profile. Importantly, the component remained strongly associated with oxygen requirements after adjustment for gestational age and birth weight, supporting the concept that respiratory requirements during HFOV-VG reflect overall cardiovascular status rather than isolated alterations in ventricular performance or pulmonary vascular loading. These findings are consistent with contemporary neonatal haemodynamic assessment frameworks that increasingly emphasise integrated physiological evaluation rather than reliance on individual echocardiographic measurements alone [1, 3, 10].

An important observation was that the cardiovascular component was independently associated with FiO₂ requirements but not with mean airway pressure, oscillatory amplitude, frequency, or DCO₂. During HFOV, continuous distending pressure influences operating lung volume, pulmonary vascular resistance, venous return, and ventricular output; both inadequate recruitment and overdistension may therefore impair cardiorespiratory coupling [30, 31]. Oxygenation depends on pulmonary gas exchange, perfusion, pulmonary vascular loading, and cardiovascular performance, whereas carbon dioxide clearance is determined predominantly by oscillatory tidal volume, frequency, and ventilatory efficiency [6, 7, 24, 32]. Individualised open-lung optimisation may have attenuated the association between mean airway pressure and the cardiovascular component; its absence should not be interpreted as evidence of no haemodynamic effect. Similar interactions may occur during other modes of mechanical ventilation, although this HFOV-VG cohort cannot establish mode specificity.

Beyond oxygenation burden, higher PC1 scores were independently associated with mortality, higher peak vasoactive–inotropic scores, longer inotropic support, and greater odds of inotrope use. VIS is an established measure of haemodynamic support intensity and has been associated with illness severity, morbidity, and mortality in critically ill neonatal and paediatric populations [14, 33–35]. These findings suggest that PC1 captures cardiovascular loading associated with haemodynamic instability and mortality; however, its prognostic value requires validation in larger independent cohorts.

The association between PC1 and mortality is consistent with previous studies linking ventricular dysfunction, pulmonary vascular disease, and impaired systemic blood flow with adverse neonatal outcomes [26, 36, 37]. Our findings extend this literature by showing that a composite cardiovascular component measured during HFOV-VG was associated with oxygenation burden, haemodynamic support, and mortality. PC1 was not independently associated with moderate-to-severe BPD, possibly because it was derived from echocardiographic measurements obtained during the first 48 h and therefore primarily reflected early transitional cardiorespiratory physiology. In contrast, BPD is determined at 36 weeks of postmenstrual age and reflects cumulative antenatal and postnatal exposures. Because serial haemodynamic assessments were unavailable, later cardiovascular changes potentially associated with BPD were not captured. Early integrated cardiovascular assessment may therefore be more relevant to acute haemodynamic instability and mortality than to later respiratory morbidity, although this interpretation requires prospective evaluation.

These findings may inform physiological assessment of preterm infants receiving HFOV-VG. Although management is primarily guided by oxygenation, blood gases, and ventilator parameters, increased oxygen requirements may also reflect cardiovascular loading and altered transitional adaptation. Targeted neonatal echocardiography could provide complementary information on ventricular performance, cardiac output, pulmonary haemodynamics, and ventricular interaction. Although PC1 cannot yet be considered a validated treatable trait, a high score may prompt comprehensive haemodynamic assessment and individualised optimisation of ventilatory settings and circulatory support, including evaluation for pulmonary vascular disease when clinically indicated. Future prospective studies should evaluate whether serial echocardiography can identify evolving cardiovascular components and whether component-guided management improves physiological or clinical outcomes [9, 38, 39]. External validation in larger multicentre cohorts is required before this approach can be incorporated into routine practice.

This study has several strengths and limitations. It combined targeted neonatal echocardiography with contemporaneous ventilator data obtained during HFOV-VG, allowing assessment of associations between cardiovascular function and respiratory requirements during the same period of respiratory support. The inclusion of myocardial deformation imaging, conventional functional indices, cardiac output measures, and markers of pulmonary vascular loading enabled evaluation of multiple cardiovascular domains. Principal component analysis was used to integrate these measurements into physiologically related cardiovascular phenotypes.

Several limitations should be acknowledged. This was a single-centre study with a modest sample size; however, management and echocardiographic acquisition were standardised within the same clinical environment. The PCA was exploratory, and its component structure requires validation in a larger independent cohort. The relatively narrow physiological distributions and modest oxygen requirements may have reduced the ability to detect associations and limit generalisability to more severely affected infants. Mean airway pressure was clinician selected but individualised using a consistent open-lung approach; consequently, its lack of association with PC1 does not exclude a haemodynamic effect. VG automatically adjusted oscillatory amplitude to maintain the targeted tidal volume, potentially attenuating ventilator-derived evidence of altered respiratory mechanics or lung volume. Echocardiography was performed at a single time point but was contemporaneous with the analysed ventilator data, allowing assessment of physiological relationships during the same period. Finally, the observational design precludes causal inference, and residual confounding remains possible despite adjustment for gestational age and birth weight. These findings should therefore be considered exploratory and validated in larger multicentre cohorts before clinical application.

## Conclusion

In preterm infants born before 30 weeks of gestation receiving HFOV-VG, the first PCA-derived cardiovascular component, integrating measures of ventricular performance, longitudinal myocardial deformation, cardiac output, and ventricular loading, was independently associated with oxygenation burden and mortality. These exploratory findings suggest that oxygen requirements during HFOV-VG may be related to cardiovascular loading and cardiorespiratory interaction as well as pulmonary physiology and require external validation.

## Supplementary Information

Below is the link to the electronic supplementary material.

ESM 1 (DOCX 27.9 KB)

ESM 2 (DOCX 27.7 KB)

## Data Availability

The datasets generated and analyzed during the current study are available from the corresponding author upon reasonable request, subject to appropriate institutional approvals.
