# Supplementary material for: Cardiorespiratory coupling during high-frequency oscillatory ventilation with volume guarantee in extremely preterm infants
Source: Eur J Pediatr. 2026 Sep 23;185(10):775. doi: 10.1007/s00431-026-07429-w (PMC13601134; doi:10.1007/s00431-026-07429-w)
Supplement: Supplementary file 1 [file 431_2026_7429_MOESM1_ESM.docx]

**Supplementary Table S1. Respiratory, cardiovascular and haemodynamic support characteristics**

| **Variable** | **Value** |
| --- | --- |
| Mean FiO₂ | 0.35 (0.31–0.52) |
| Mean MAP, mbar | 10.6 (9.8–11.8) |
| Mean oscillatory amplitude, mbar | 27.5 (25.0–30.0) |
| Mean oscillatory frequency, Hz | 15.0 (15.0–17.0) |
| DCO₂ | 35.5 (29.6–52.6) |
| Duration of invasive ventilation, days | 9.5 (6.0–19.2) |
| Left ventricular output, mL/kg/min | 260 (205–320) |
| Left ventricular ejection fraction, % | 61 (56.5–64.5) |
| Left ventricular fractional shortening, % | 35 (34–36) |
| Left ventricular strain | −20.0 (−21.0 to −19.5) |
| Right ventricular output, mL/kg/min | 310 (285–345) |
| TAPSE, mm | 6.0 (5.0–7.0) |
| Right ventricular fractional area change, % | 45 (44.5–46.0) |
| Right ventricular strain | −20.0 (−20.0 to −19.0) |
| PAAT/RVET ratio | 0.26 (0.23–0.30) |
| Eccentricity index | 1.1 (1.1–1.2) |
| Any inotrope exposure | 32/62 (51.6%) |
| Peak VIS | 6.0 (0–45) |
| Duration of inotropes, days | 3.0 (2.0–6.5) |
| Haemodynamically significant PDA | 27/62 (43.5%) |

Values are presented as median (interquartile range) or n/N (%), as appropriate. DCO₂, carbon dioxide diffusion coefficient; FiO₂, fraction of inspired oxygen; MAP, mean airway pressure; PAAT/RVET, pulmonary artery acceleration time-to-right ventricular ejection time ratio; PDA, patent ductus arteriosus; TAPSE, tricuspid annular plane systolic excursion; VIS, vasoactive–inotropic score.
