# Supplementary material for: Cardiorespiratory coupling during high-frequency oscillatory ventilation with volume guarantee in extremely preterm infants
Source: Eur J Pediatr. 2026 Sep 23;185(10):775. doi: 10.1007/s00431-026-07429-w (PMC13601134; doi:10.1007/s00431-026-07429-w)
Supplement: Supplementary file 2 [file 431_2026_7429_MOESM2_ESM.docx]

**Supplementary Table S2. Correlations between echocardiographic measures and oxygenation burden during HFOV-VG**

| **Echocardiographic measure** | **Spearman rho (95% CI)** | **Strength** | **p value** | **q value** |
| --- | --- | --- | --- | --- |
| RV strain | 0.53 (0.32 to 0.69) | Moderate | <0.001 | <0.001 |
| LVEF | −0.40 (−0.59 to −0.17) | Moderate | 0.001 | 0.005 |
| Eccentricity index | 0.40 (0.16 to 0.59) | Moderate | 0.002 | 0.005 |
| LVFS | −0.36 (−0.56 to −0.12) | Weak | 0.004 | 0.010 |
| LV strain | 0.35 (0.11 to 0.55) | Weak | 0.006 | 0.010 |
| RV FAC | −0.35 (−0.55 to −0.10) | Weak | 0.006 | 0.010 |
| RVO | −0.28 (−0.49 to −0.03) | Weak | 0.032 | 0.039 |
| TAPSE | −0.28 (−0.50 to −0.03) | Weak | 0.030 | 0.039 |
| LVO | −0.24 (−0.47 to 0.01) | Weak | 0.060 | 0.064 |
| PAAT/RVET | −0.24 (−0.46 to 0.01) | Weak | 0.064 | 0.064 |

CI, confidence interval; FAC, fractional area change; LV, left ventricular; LVEF, left ventricular ejection fraction; LVFS, left ventricular fractional shortening; LVO, left ventricular output; PAAT/RVET, pulmonary artery acceleration time-to-right ventricular ejection time ratio; RV, right ventricular; RVO, right ventricular output; TAPSE, tricuspid annular plane systolic excursion.
